# Robot-Assisted Resection of Mediastinal Tumors in Pediatric Patients: A Systematic Review

**DOI:** 10.3390/children13070937

**Published:** 2026-07-17

**Authors:** Donatella Di Fabrizio, Irene Tavolario, Francesca Mastroberti, Piero Canzio, Edoardo Bindi, Giovanni Cobellis

**Affiliations:** 1 Pediatric Surgery Unit, Salesi Children’s Hospital, 60123 Ancona, Italy; irene.tavolario@ospedaliriuniti.marche.it (I.T.); francesca.mastroberti@ospedaliriuniti.marche.it (F.M.); edoardo.bindi@ospedaliriuniti.marche.it (E.B.); giovanni.cobellis@ospedaliriuniti.marche.it (G.C.); 2 Department of Specialized Clinical and Odontostomatological Sciences, University Politecnica of Marche, 60126 Ancona, Italy

**Keywords:** robotic surgery, mediastinal tumor, mediastinal lesions, pediatric, children

## Abstract

**Introduction:** Mediastinal tumors in children are rare and heterogeneous, and their surgical management is challenging due to limited working space and proximity to critical structures. While thoracoscopy has expanded, technical limitations persist in complex mediastinal dissections. Robot-assisted surgery may address these limitations, but its role in pediatric mediastinal tumor resection is not well defined. This systematic review aims to synthesize the available evidence on robot-assisted resection of mediastinal tumors in children, focusing on patient selection, technical feasibility, perioperative outcomes, and available oncologic results. **Methods:** A systematic review was conducted in accordance with PRISMA 2020 guidelines and registered in PROSPERO (CRD420251245437). PubMed/MEDLINE, Web of Science, Scopus, and the Cochrane Library were searched up to 1 December 2025. Eligible studies included pediatric patients (0–18 years) undergoing robot-assisted resection of mediastinal tumors. Study selection, data extraction, and risk of bias assessment were performed independently by two reviewers. Due to heterogeneity and lack of comparative data, a qualitative narrative synthesis was undertaken. **Results:** Fifteen studies were included, comprising retrospective case series and case reports, including 215 children, 149 (69.3%) of whom were derived from a single retrospective study. The most frequent indications were localized neurogenic tumors, predominantly in the posterior mediastinum. Robot-assisted surgery was consistently performed with curative intent. The da Vinci platform was used in all cases, with reproducible port-placement strategies adapted to tumor location and patient size. Conversion to open surgery occurred in 11 (5.1%) cases, mainly due to bleeding or limited exposure, with no emergency conversions reported. Reported perioperative complications were uncommon, although complication reporting was heterogeneous across studies. No perioperative deaths were reported. Two disease-related deaths occurred during follow-up in patients with recurrent Ewing sarcoma. Long-term oncologic outcomes could not be adequately assessed because of heterogeneous reporting and limited follow-up. **Conclusions:** Robot-assisted resection of mediastinal tumors in children appears feasible in carefully selected cases treated at experienced centers. Current evidence mainly supports its use for localized tumors with favorable anatomy, particularly in the posterior mediastinum, while long-term oncologic adequacy remains insufficiently defined.

## 1. Introduction

Mediastinal tumors in the pediatric population are rare and heterogeneous, encompassing a wide spectrum of pathologies such as neurogenic tumors, germ cell tumors, thymic neoplasms, and other solid mediastinal masses [1,2]. Surgical management is often challenging due to the confined thoracic space, proximity to critical structures, and the small anatomical dimensions of pediatric patients [3]. While open thoracotomy has traditionally represented the standard approach, minimally invasive techniques have progressively gained acceptance in selected cases, aiming to reduce surgical trauma and postoperative morbidity [4,5].

Thoracoscopic surgery has expanded the minimally invasive armamentarium in pediatric thoracic oncology; however, limitations related to two-dimensional vision, restricted instrument articulation, and ergonomic constraints remain in complex mediastinal dissections [6,7]. Robot-assisted surgery has been proposed as an evolution of minimally invasive surgery, offering three-dimensional magnified visualization, tremor filtration, and enhanced dexterity through articulated instruments [8,9,10]. Although these characteristics may facilitate complex mediastinal dissection, their potential technical advantages have not yet been shown to translate into superior clinical or oncologic outcomes in pediatric patients. These features may be particularly advantageous in the pediatric mediastinum, where precise dissection and preservation of vital structures are paramount [11].

Despite the increasing diffusion of robotic platforms in pediatric urology and abdominal surgery, the application of robot-assisted techniques to mediastinal tumor resection in children remains limited and fragmented. The available literature consists predominantly of small retrospective case series and isolated case reports, with heterogeneous reporting of indications, surgical techniques, and outcomes. To the best of our knowledge, no previous systematic review has specifically focused on robot-assisted resection of mediastinal tumors in the pediatric population, leaving the current evidence on patient selection, technical feasibility, perioperative outcomes, and oncologic adequacy insufficiently defined.

The aim of this systematic review was to synthesize the available evidence on robot-assisted resection of mediastinal tumors in pediatric patients by describing patient selection, surgical indications, technical characteristics, perioperative outcomes, and the available oncologic results. Given the absence of comparative studies, this review was designed to provide a descriptive synthesis of the published experience.

## 2. Methods

### 2.1. Study Design and Protocol Registration

This systematic review was conducted in accordance with the Preferred Reporting Items for Systematic Reviews and Meta-Analyses (PRISMA) 2020 guidelines [12]. The review methodology was defined a priori and followed throughout all stages of the study. The protocol was prospectively registered in the PROSPERO database (CRD420251245437).

### 2.2. Eligibility Criteria

Studies were selected based on eligibility criteria regarding population, intervention, outcomes, study design, and setting.

The population of interest included pediatric patients aged 0–18 years diagnosed with mediastinal tumors of any histology, including neuroblastic tumors, germ cell tumors, thymomas, ganglioneuromas, ganglioneuroblastomas, and other solid mediastinal masses. Studies enrolling mixed pediatric and adult populations were eligible only if pediatric data were reported separately or could be extracted independently. Adult-only cohorts and studies addressing non-tumoral mediastinal conditions, such as cysts or thymectomy for myasthenia gravis without neoplastic disease, were excluded.

The intervention of interest was robot-assisted surgery for mediastinal tumor resection, performed using any robotic platform and technique. Studies reporting exclusively non-robotic surgical approaches, including open or thoracoscopic surgery without robotic data, were excluded. A comparator group was not required for inclusion.

Eligible studies were required to report at least one perioperative and postoperative outcome related to feasibility, safety, or clinical results. Outcomes of interest included operative technical details, operative time, estimated intraoperative blood loss when available, conversion to thoracoscopic or open surgery and reasons for conversion, postoperative complications, length of hospital stay, recurrence, mortality, and follow-up.

Original clinical studies were eligible for inclusion, including prospective or retrospective cohort studies, case series, and case reports with extractable data. Reviews, meta-analyses, editorials, letters, expert opinions, conference abstracts without sufficient data, technical notes without patient-level outcomes, animal or cadaveric studies, and simulation-based reports were excluded. Only human studies published in English were considered. No restrictions were applied regarding country of origin, healthcare system, or clinical setting.

### 2.3. Information Sources and Search Strategy

A systematic literature search was performed in PubMed/MEDLINE, Web of Science, Scopus, and the Cochrane Library. No restrictions on publication date were applied. The search was conducted up to 1 December 2025.

The search strategy combined controlled vocabulary terms (Medical Subject Headings, MeSH) and free-text keywords related to robotic surgery, mediastinal tumors, and pediatric populations. Search terms included variations in robotic surgery, robot-assisted, mediastinal neoplasms, thoracic tumors, and pediatric-related terms such as child, infant, adolescent, pediatric, and paediatric. Equivalent search strategies were adapted to the syntax and indexing systems of each database. Reference lists of all included articles were manually screened to identify additional eligible studies.

### 2.4. Study Selection

All records retrieved from the database searches were imported into reference management software [13], and duplicate records were removed prior to screening. Titles and abstracts were independently screened by four reviewers (DDF, IT, FM, PC) to identify potentially eligible studies. Full texts of all records deemed potentially relevant were retrieved and independently assessed against the predefined eligibility criteria.

Reasons for exclusion at the full-text stage were recorded and categorized. Any disagreements between reviewers at any stage of the screening and selection process were resolved through discussion and consensus. Potential overlap between studies from the same institution and study period was assessed by comparing recruitment periods, participating centers, patient characteristics, and reported cohorts. When overlap was suspected, the most comprehensive dataset was retained for qualitative synthesis.

### 2.5. Data Extraction

Data extraction was performed using a standardized data collection form. One reviewer (DDF) extracted data from each included study, and the extracted information was checked for accuracy and completeness by a second reviewer (EB).

Extracted data included study characteristics (author, year of publication, country, and study design), patient demographics (age, weight when available, comorbidities or syndromic conditions), and tumor characteristics (histology, mediastinal compartment, maximum tumor diameter, and multifocality).

Surgical and perioperative variables included robotic platform, surgical approach and side, patient positioning, number and size of robotic and assistant ports, ventilation strategy, CO_2_ insufflation pressure, docking time and console time when reported, operative time, estimated blood loss, need for transfusion, and conversion defined as the need to abandon the planned robotic procedure in favor of thoracoscopic or open surgery. Postoperative and oncological outcomes included postoperative complications, including all adverse events reported by the original authors during hospital stay or follow-up and classified according to the Clavien–Madadi classification [14,15] when reported, chest drain use and duration, reintervention, length of hospital stay, 30-day readmission, mortality, and duration of follow-up. Oncologic outcomes included recurrence, disease progression, resection margins when available, and disease-related mortality. Missing or unreported data were recorded as not available.

### 2.6. Risk of Bias Assessment

The methodological quality and risk of bias of included studies were assessed using the Joanna Briggs Institute (JBI) Critical Appraisal Tools [16], selected according to study design. The JBI checklist for case reports was applied to single-patient studies, while the JBI checklist for case series was used for non-comparative observational studies.

Risk of bias assessment was performed independently by two reviewers (EB and DDF), with disagreements resolved by consensus. Risk of bias due to missing results, including publication bias or selective outcome reporting, was not formally assessed, as the included evidence consisted predominantly of case reports and small case series, for which statistical methods to evaluate reporting bias are not applicable.

### 2.7. Data Synthesis

Given the clinical and methodological heterogeneity of the included studies and the absence of comparative outcome data, a qualitative narrative synthesis was performed. Quantitative pooling and meta-analysis were not planned.

Data were summarized descriptively and organized into structured tables. For continuous variables reported by more than one study, weighted means were calculated using the number of patients contributing to each study as weights and including only studies reporting the variable of interest (outcome-specific denominators). No estimates of variance were calculated because individual patient-level data were not available. Categorical variables were reported as absolute numbers when applicable. Technical aspects of the robotic approach were synthesized qualitatively, focusing on patient positioning, port configuration, robotic platform, docking and console times, and reported technical limitations or adaptations. The strengths and limitations of the available evidence were interpreted in light of study design, sample size, and risk of bias.

## 3. Results

**Figure 1:**
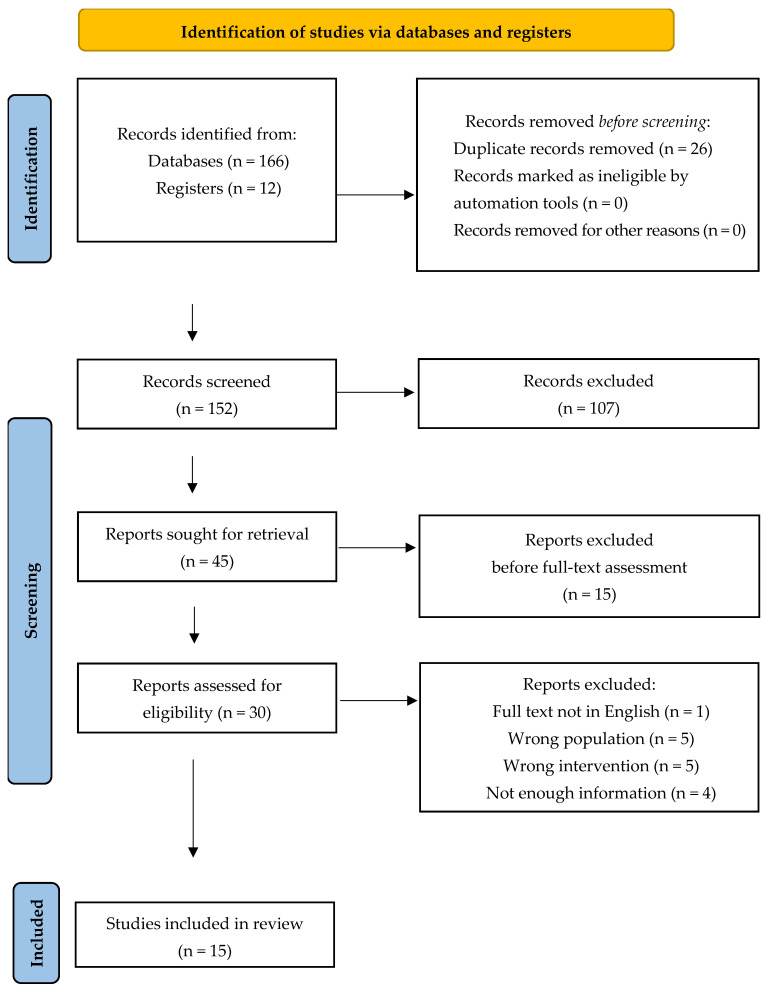
Prisma 2020 methodology for systematic review.

The systematic search identified 178 records, including 166 from electronic databases and 12 from registers. After the removal of 26 duplicates, 152 records were screened by title and abstract, and 107 were excluded. Forty-five full-text articles were sought for retrieval. Fifteen reports were excluded after retrieval because they did not meet the predefined eligibility criteria, leaving 30 full-text articles for eligibility assessment. Fifteen studies were excluded: full text not available in English (*n* = 1), wrong population (*n* = 5), wrong intervention (*n* = 5), and insufficient data (*n* = 4). Ultimately, 15 studies [17,18,19,20,21,22,23,24,25,26,27,28,29,30,31] met eligibility criteria and were included in the qualitative synthesis. The selection process is summarized in the PRISMA 2020 flow diagram (Figure 1).

The first reports of robot-assisted surgery for pediatric mediastinal tumors appeared in 2008 [17,25]. No further publications were identified for more than a decade, until 2022. Since then, renewed interest has emerged. Most studies originated from Asia (46%), mainly China and Japan, followed by the United States (27%), Europe (20%), and Morocco (7%) (Table 1).

All studies were observational and included retrospective case series and case reports, reflecting the rarity of the condition and the highly specialized nature of the procedure. Overall, the studies reported outcomes for 215 pediatric patients undergoing robot-assisted mediastinal tumor surgery. One retrospective single-center study contributed 149 patients (69.3% of the overall cohort), whereas the remaining studies consisted of small retrospective series or isolated case reports. Consequently, the available evidence largely reflects the experience of a single institution.

Among case series, methodological quality was acceptable, with clear inclusion criteria, valid case identification, standardized clinical measurements, and well-reported perioperative and follow-up outcomes; consecutive or complete inclusion was not explicitly stated in a minority of studies. Case reports consistently described patient characteristics, diagnostic workup, surgical procedures, and post-intervention outcomes, and all provided clear clinical take-home messages, although adverse events were infrequently reported. However, the overall certainty of the evidence remains limited by the predominance of case reports and small retrospective case series, with inherent risks of selection bias. Comparative effectiveness and long-term oncological outcomes could not be assessed due to the non-comparative design of the included studies.

### 3.1. Patient and Tumor Characteristics

Across the included studies, robot-assisted surgery was performed in 215 children and adolescents, with individual study sample sizes ranging from 1 to 149 patients. Age data were available for 201 patients and ranged from 6 months to 17 years, with a weighted mean age of 6.2 years. Body weight was reported for 195 patients and ranged from 8 to 72 kg, with a weighted mean of 24.1 kg, showing that robotic mediastinal surgery has been applied across a broad pediatric age and weight range (Table 1). No relevant comorbidities were reported in the included studies.

Tumor histology showed predominance of neurogenic tumors (*n* = 145). Other subtypes included thymic tumors (*n* = 19), foregut-derived lesions (*n* = 19), mesenchymal tumors (*n* = 8), vascular and lymphatic tumors (*n* = 9), lipogenic tumors (*n* = 6), germ cell tumors (*n* = 5), neuroendocrine tumors (*n* = 2), and lesions of unclear histology (*n* = 2). This distribution reflects the spectrum of tumors reported in the available literature and the apparent preference for selecting well-demarcated lesions for robotic surgery. Tumors were most often located in the posterior mediastinum, followed by selected anterior mediastinal lesions, suggesting that posterior mediastinal tumors represent the most common indication for robotic resection (Table 2).

Reporting of neoadjuvant therapy was inconsistent. Reported in 199 patients, the mean tumor diameter was 11.5 cm (range 5–12.6 cm). Most cases involved localized tumors without major vascular invasion, indicating a trend toward careful preoperative selection of anatomically favorable lesions for a robotic approach.

### 3.2. Indications for a Robotic Approach

Across the included studies, the primary indication for surgery was radical resection, which was consistently pursued whenever complete tumor removal was deemed oncologically appropriate. Diagnostic biopsy or debulking procedures were not reported, reflecting a general preference for definitive surgical management in selected patients.

Indications for a robot-assisted approach were largely based on a combination of tumor-related and patient-related factors rather than on standardized criteria [18,20,22,24]. The most frequently cited determinants included tumor size and localization, absence of major vascular encasement, and surgeon and institutional experience with robotic systems [20,22]. In several studies, the choice of a robotic approach was explicitly described as a matter of surgical preference [17,19,21,23,25,26,27,28,29,30,31], while others reported a structured decision-making process integrating preoperative imaging, assessment of surgical risk factors, and multidisciplinary tumor board discussion [18,20,22,24].

Age and body weight were occasionally considered, with some authors limiting robotic indications to children older than 6 months and weighing more than 8 kg [18], whereas others reported no strict size- or weight-based exclusion criteria. Conversely, patients requiring an open approach due to anticipated technical complexity were generally excluded from robotic surgery [18].

More explicit selection criteria were proposed by Blanc et al. [22], who suggested considering robotic thoracic surgery for paravertebral neuroblastomas, tumors confined to the thymic bed (such as teratomas and thymomas), and isolated pulmonary metastasectomy, while identifying age below 2 years, vascular encasement, and extension to the median mediastinum (including pericardium, esophagus, or trachea) as contraindications.

### 3.3. Surgical and Technical Characteristics

**Figure 2:**
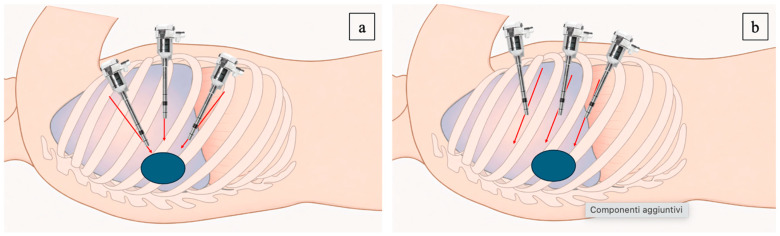
Trocar placement strategies for robot-assisted resection of pediatric mediastinal tumors. (**a**) Triangulated position; (**b**) parallel position.

Despite inter-institutional variability, technical patterns recurred. The da Vinci platform was used in all cases, with newer systems adopted over time. Trocar placement varied but converged toward configurations dictated by tumor location, patient size, and collision avoidance. For multiport systems, two concepts emerged. Most frequent was a triangulated configuration, with camera and working ports forming a triangle centered on the lesion [19,20,26,27,28,29] (Figure 2a). This approach was used for posterior and paraspinal tumors, enabling triangulation and depth perception while preserving interport distance of at least one hand-width (7–8 cm) to reduce arm interference.

The second strategy was a parallel-line configuration, in which robotic ports were aligned along vertical or oblique lines according to tumor location rather than fixed intercostal spaces [18] (Figure 2b). This technique, particularly described in larger series, aimed to maintain a minimum distance of 3 cm between ports while adapting trocar placement to different mediastinal compartments, especially for anterior and upper posterior lesions. Patient positioning was closely integrated with trocar strategy. Posterior and paraspinal tumors were generally approached with the patient in lateral decubitus position, whereas anterior mediastinal lesions were more frequently addressed using a modified supine or semi-lateral position, facilitating anterior access and visualization. In all configurations, trocar placement was individualized based on preoperative imaging, emphasizing the importance of tailoring port geometry to tumor anatomy rather than adhering to rigid intercostal landmarks.

Kaneda et al. [31] described a distinct single-port robotic approach through a single subcostal incision, avoiding intercostal spaces. This allowed multiple articulated instruments through one access point while preserving three-dimensional visualization and wristed instrumentation. The subcostal route was advantageous for large posterior mediastinal neurogenic tumors, reducing potential intercostal nerve injury and long-term thoracic wall deformity in growing children.

### 3.4. Operative Parameters and Intraoperative Management

Intraoperative parameters were variably reported across studies, reflecting differences in size, tumor and practice, yet several consistent patterns emerged (Table 3). When specified, carbon dioxide insufflation pressures were kept deliberately low, ranging from 2 to 8 mmHg, with lower pressures preferentially adopted in smaller children to minimize cardiopulmonary compromise while preserving adequate operative exposure. Single-lung ventilation was routinely employed, most commonly achieved using a double-lumen endotracheal tube, whereas single-lumen tubes with bronchial blockers were used in smaller patients, underscoring the need for anesthetic strategies tailored to patient age and size [20,25,27,29,30,31].

Docking time was infrequently reported but, when available, ranged from 3 to 45 min, while console time showed wider variability (42.5–294 min), reflecting differences in tumor complexity and surgeon experience. Total operative time similarly ranged broadly, from approximately 87 to 386 min, with longer procedures generally associated with larger tumors, complex dissections, or early phases of the learning curve [17,18,19,20,21,23,24,28,30,31].

Tumor retrieval was most commonly performed using an endoscopic retrieval bag, with specimen extraction achieved either through the original trocar site or by modest enlargement of the incision, typically to 2–5.5 cm, depending on tumor size and consistency [17,18,19,22,27,28,29,30,31]. Importantly, Meehan et al. [17] emphasized technical adaptations to optimize instrument articulation in small thoracic cavities, such as partial withdrawal of the trocar to position the remote center just outside the chest wall, thereby increasing effective intrathoracic working length.

When reported, estimated blood loss was low across the included studies, and intraoperative transfusion was rarely required [19].

Overall, 11 (5.1%) conversions to open surgery were reported [18,19,20,22]. Reasons included bleeding, poor visualization, invasion of adjacent structures, restricted working space, or dense adhesions. No emergency conversions were described. Multiple authors emphasized conversion as part of a safe robotic strategy, especially when oncological principles were at risk [21,22]. Conversion rates decreased with surgeon experience and improved selection, supporting recommendations to begin with smaller, well-circumscribed tumors and maintain a low threshold for conversion during difficult dissection [22].

### 3.5. Postoperative Outcomes

When specified, a chest drain was routinely placed, with drainage duration ranging from 1 to 20 days, most commonly between 1 and 4 days [17,18,20,25,27,28,31]. Mean postoperative hospital stay ranged from 1.4 to 8.9 days, with longer admissions generally associated with postoperative complications or prolonged drainage [17,18,19,20,21,23,25,28,29,31].

No perioperative deaths were reported. During follow-up, two patients died because of disease progression after recurrence of Ewing sarcoma rather than as a consequence of the surgical procedure [20]. In-hospital complications included chylous effusion [20], pneumothorax [22], transient intraoperative hypertension [23], and transient right phrenic nerve paralysis [30], most of which were managed conservatively or with minor interventions. Outpatient complications comprised respiratory distress secondary to chyle leak requiring drainage repositioning [19] and during follow-up, tumor recurrence with pulmonary nodules requiring surgical resection was reported in one patient [30]. According to the Clavien–Madadi classification, reported complications reached up to grade III, with reoperation required for recurrence [30], drainage repositioning [19,22], or completion thymectomy following partial resection [22]. Because resection margins, lymph node assessment, recurrence definitions, and follow-up duration were inconsistently reported, no conclusions regarding oncologic adequacy or oncologic equivalence of the robotic approach can be drawn from the available evidence.

## 4. Discussion

This systematic review provides the most comprehensive synthesis to date of robot-assisted surgery for mediastinal tumors in pediatric patients. By integrating data from more than two decades of published experience, it delineates the clinical scenarios in which robotic surgery has been applied, clarifies technical patterns, and contextualizes perioperative and oncologic outcomes within the constraints of a rare and heterogeneous disease setting.

A first key finding is that robotic surgery has been almost exclusively employed with a curative intent, as radical resection was the predominant indication across studies [17,18,19,20,21,22,23,24,25,26,27,28,29,30,31]. Diagnostic biopsy or debulking was not reported, underscoring that the robotic platform has been preferentially adopted when complete resection was considered oncologically appropriate. This reflects a cautious and selective use of robotics in children, where technical feasibility is consistently subordinated to oncological principles.

Patient and tumor selection emerged as a central theme [18,20,22,24]. Although formal inclusion criteria were inconsistently reported, a convergent pattern was evident: robotic surgery was mainly applied to localized mediastinal tumors with favorable anatomy, particularly neurogenic lesions of the posterior mediastinum and selected anterior mediastinal tumors. Tumor size alone did not represent a strict limiting factor; rather, tumor location, absence of major vascular encasement, and institutional experience appeared to drive decision-making [20,22]. This implicit consensus suggests that feasibility and safety, rather than rigid dimensional thresholds, currently define candidacy for robotic resection.

Technically, variability in trocar placement and setup reflects adaptation to pediatric anatomy rather than a lack of standardization. Two reproducible concepts, triangulated port placement [19,20,26,27,28,29] and parallel-line configurations [18], were consistently reported and tailored to different mediastinal compartments. Single-port subcostal robotic access further expands the technical options for posterior tumors and may reduce intercostal trauma in growing children [31,32,33]. These strategies emphasize imaging-based planning and flexibility over rigid port maps.

The available perioperative data suggest that robot-assisted mediastinal surgery is technically feasible in carefully selected pediatric patients treated at experienced centers. Operative time and length of stay varied across studies and reflected differences in patient selection, tumor characteristics, and institutional experience [11]. Reported perioperative complications were uncommon, although complication reporting was inconsistent across studies. Conversions to open surgery occurred in a limited number of cases and were consistently reported as elective decisions driven by bleeding, limited exposure, or concerns regarding oncologic radicality [18,19,20,22]. Rather than representing failure, conversion emerged as an integral safety mechanism, particularly during early experience, reinforcing the concept that robotic surgery should be embedded within a hybrid minimally invasive strategy.

Oncologic outcomes should be interpreted cautiously. Disease recurrence and disease-related deaths were reported in patients with aggressive tumor biology [20], whereas reporting of resection margins, completeness of resection, lymph node assessment, and duration of follow-up was inconsistent across studies. Consequently, the available evidence does not permit conclusions regarding the oncologic adequacy or equivalence of the robotic approach. Tumor biology undoubtedly influences prognosis, although completeness of resection and appropriate patient selection also remain important determinants of oncologic outcome.

This review has limitations. Most pooled patients came from a single retrospective series, which disproportionately influences the distribution of indications, technical patterns, conversions, and perioperative outcomes across the included studies. The evidence comes exclusively from non-comparative observational studies, with heterogeneous reporting and variable follow-up. Publication bias should also be considered, as successful robotic procedures are more likely to be reported than unsuccessful experiences. Therefore, this synthesis describes the published experience rather than a balanced estimate of practice across centers. These limitations reflect the rarity of pediatric mediastinal tumors and the ethical and practical barriers to comparative trials. Despite these limitations, the available literature provides a useful overview of the current application of robot-assisted surgery for selected pediatric mediastinal tumors and highlights the need for standardized reporting and prospective multicenter collaboration.

## 5. Conclusions

In conclusion, robot-assisted resection of mediastinal tumors in children appears technically feasible in selected patients treated at experienced centers and guided by meticulous preoperative planning. Its role appears most clearly defined for localized tumors with favorable anatomy, particularly in the posterior mediastinum. Future efforts should focus on standardized reporting of oncologic variables, longer follow-up, and collaborative multicenter registries to better define the long-term oncologic impact and refine patient selection criteria.

## Figures and Tables

**Table 1:** Demographics.

	Study						Population				
	First Author	Year of Publication	Journal	Country	Study Period	Number of Centers	Patients	Age Mean (Years)	Age Range (Years)	Weight Mean (kg)	Weight Range (kg)
Retrospective Study	Meehan J et al.	2008	J Laparoendosc Adv Surg Tech A	USA	N/A	Single center	5	9.8	2–17	41.5	13.9–70.5
	Zeng Q et al.	2023	Eur J Cardiothorac Surg	China	March 2021–November 2022	Single center	149	5.9	6 mo–16 y	23.6	8.0–72.0
	Svetanoff WJ et al.	2024	J Pediatr Surg	USA	December 2015–March 2023	Multicenter	17	6.1	4.5–8.8	22.2	15.9–30
	Palo F et al.	2025	Front Pediatr	Italy	2020–2025	Single center	20	5	16 mo–17 y	25	11.0–72.0
Case series	Vatta F et al.	2022	Front Pediatr	Italy	2012–2021	Single center	1	7.6	/	N/A	/
	Blanc T et al.	2022	Ann Surg Oncol	France	2016–2020	Multicenter	14	N/A	N/A	N/A	N/A
	Bahadir GB et al.	2023	Turk J Pediatr Dis	Turkey	November 2017–April 2022	Single center	1	11	/	30	/
	El Mohady B et al.	2025	Indian J Surg Oncol	Morocco	June 2014–June 2023	Single center	1	12	/	39.5	/
Case Report	DeUgarte D et al.	2008	J Laparoendosc Adv Surg Tech A	USA	/	Single center	1	17	/	N/A	/
	Nemoto Y et al.	2022	Surg Case Rep	Japan	/	Single center	1	15	/	N/A	/
	Ochi T et al.	2023	Asian J Endosc Surg	Japan	/	Single center	1	2	/	11	/
	Prasad A et al.	2023	J Pediatr Endosc Surg	India	/	Single center	1	9	/	N/A	/
	Hanke R et al.	2024	J Surg Case Rep	USA	/	Single center	1	10	/	N/A	/
	Fukushima Y et al.	2024	Multimed Man Cardiothorac Surg	Japan	/	Single center	1	16	/	N/A	/
	Kaneda S et al.	2025	JTCVS Tech	Japan	/	Single center	1	8	/	22.5	/

**Table 2:** Tumor histology.

Tumor Category	Number
Neurogenic tumors	145
Thymic tumors	19
Foregut-derived lesions	19
Vascular/lymphatic tumors	9
Mesenchymal/soft tissue tumors	8
lipogenic tumors	6
Germ cell tumors	5
Neuroendocrine tumors	2
Inflammatory/unclear etiology	2
Total	215

**Table 3:** Operative and postoperative outcome.

	Operative Management									Postoperative Outcomes						
Author	Robotic Platform	Patient Position	Number of Robotic Ports	Robotic Port Sizes (mm)	Assistant Port Sizes (mm)	CO_2_ Insufflation Pressure (mmHg)	Tumor Retrieval	Conversion (Number, Open/VATS)	Reason for Conversion	Chest Drain (Days)	LOS (Days—Mean, Range)	Mortality	In-Hospital Complications	Outpatient Complication	Clavien–Madadi Grade	Reoperation
Meehan J et al.	da Vinci S	Lateral	3	5	5	N/A	Endobag	0	/	1 to 3	1.4	0	0	0	0	0
Zeng Q et al.	da Vinci Xi	Lateral	3	8	5	N/A	Endobag (1.5–2 cm incision)	4, open	Bleeding, tumor invasion	2 to 3	7.2 (4.0–14.0)	0	0	0	0	0
Svetanoff WJ et al.	da Vinci Si	Lateral	3	8	0	N/A	Endobag	2, open	Poor visualisation, bleeding	N/A	1.5	0	0	Respiratory distress and hydropneumothorax secondary to chyle leak (1) (drainage positioning)	3a	0
Palo F et al.	da Vinci Xi	Lateral	3 or 4	8	0	2 to 6	N/A	4, open	Difficulties in manipulating the tumor, adhesion	1 to 20	8.9	Ewing sarcoma recurrence and progression (2)	Chylous effusions (2) (prolonged drainage)	0	2	0
Vatta F et al.	da Vinci Si	Lateral	3	8	5	N/A	N/A	0		N/A	7	0	0	0	0	0
Blanc T et al.	N/A	N/A	3	8	5	4	Endobag (enlarged incision)	1, open	Narrow space	N/A	N/A	0	Pneumothorax (2) (drain)	0	3a	Partial thymectomy to complete thymectomy (1)
Bahadir GB et al.	da Vinci Si	Lateral	3	8	10	N/A	N/A	0		N/A	7	0	Intraoperative hypertension (1)	0	/	0
El Mohady B et al.	da Vinci Si	Lateral	3	8	5	N/A	N/A	0		N/A	N/A	0	0	0	0	0
DeUgarte D et al.	da Vinci Si	Lateral	3	8	N/A	N/A	N/A	0	/	4	4	0	0	0	0	0
Nemoto Y et al.	da Vinci Xi	Lateral	4	8	0	8	N/A	0	/	N/A	N/A	0	0	0	0	0
Ochi T et al.	da Vinci Xi	Lateral	3	8	12	4	Enlarged incision (12 mm to 3 cm)	0	/	2	N/A	0	0	0	0	0
Prasad A et al.	da Vinci Xi	Lateral with modified prone position	3	8	5	6 to 8	Endobag	0	/	1	2	0	0	0	0	0
Hanke R et al.	N/A	Lateral	3	8	5 and 12	N/A	Endobag (12 mm to 1 cm)	0	/	/	3	0	0	0	0	0
Fukushima Y et al.	da Vinci Xi	Supine (Subxiphoid approach)	4	8	5	8	Endobag	0	/	/	N/A	0	Transient right phrenic nerve paralysis (1)	Recurrence, lung nodules (1) (resection)	1b, 3b	1
Kaneda S et al.	da Vinci SP	Lateral subcostal approach	1	25	0	8	Enlarged incision	0	/	1	3	0	0	0	0	0

## Data Availability

The data underlying this article are available in the article and in its online Supplementary Material.

## Supplementary Materials

The following supporting information can be downloaded at https://www.mdpi.com/article/10.3390/children13070937/s1.

## Abbreviations

PRISMA	Preferred Reporting Items for Systematic Reviews and Meta-Analyses
PROSPERO	International Prospective Register of Systematic Reviews
MeSH	Medical subject headings
CO_2_	Carbon dioxide
JBI	Joanna Briggs Institute
N/A	Not available
mm	Millimeters
mmHg	Millimeters of mercury
LOS	Length of stay
kg	Kilograms
mo	Months
y	Years
PMID	PubMed identifier
PMCID	PubMed central identifier
VATS	Video-assisted thoracic surgery
RATS	Robot-assisted thoracoscopic surgery
