# Supplementary material for: Robot-Assisted Resection of Mediastinal Tumors in Pediatric Patients: A Systematic Review
Source: Children (Basel). 2026 Jul 17;13(7):937. doi: 10.3390/children13070937 (PMC13406298; doi:10.3390/children13070937)
Supplement: Supplementary file 1 [file children-13-00937-s001.zip › risk of bias evaluation.pdf]

Supplemental Material: Risk of Bias

	Were there clear criteria for inclusion in the case series?	Was the condition measured in a standard, reliable way for all participants included in the case series?	Were valid methods used for identification of the condition for all participants included in the case series?	Did the case series have consecutive inclusion of participants?	Did the case series have complete inclusion of participants?	Was there clear reporting of the demographics of the participants in the study?	Was there clear reporting of clinical information of the participants?	Were the outcomes or follow up results of cases clearly reported?	Was there clear reporting of the presenting site(s)/clinic(s) demographic information?	Was statistical analysis appropriate?
Meehan J et al	● (green)	● (green)	● (green)	● (green)	● (green)	● (green)	● (green)	● (green)	● (green)	● (green)
Zeng Q et al	● (green)	● (green)	● (green)	● (green)	● (green)	● (green)	● (green)	● (green)	● (green)	● (green)
Svetanoff WJ et al	● (green)	● (green)	● (green)	● (green)	● (green)	● (green)	● (green)	● (green)	● (green)	● (green)
Palo F et al	● (green)	● (green)	● (green)	● (green)	● (green)	● (green)	● (green)	● (green)	● (green)	● (green)
Vatta F et al	● (green)	● (green)	● (green)	● (green)	● (green)	● (green)	● (green)	● (green)	● (green)	● (green)
Blanc T et al	● (green)	● (green)	● (green)	● (green)	● (green)	● (green)	● (green)	● (green)	● (green)	● (green)
Bahadir GB et al	● (green)	● (green)	● (green)	● (green)	● (green)	● (green)	● (green)	● (green)	● (green)	● (green)
El Mohady B et al	● (green)	● (green)	● (green)	● (grey)	● (grey)	● (green)	● (green)	● (green)	● (green)	● (green)

	Were patient's demographic characteristics clearly described?	Was the patient's history clearly described and presented as a timeline?	Was the current clinical condition of the patient on presentation clearly described?	Were diagnostic tests or methods and the results clearly described?	Was the intervention(s) or treatment procedure(s) clearly described?	Was the post-intervention clinical condition clearly described?	Were adverse events (harms) or unanticipated events identified and described?	Does the case report provide takeaway lessons?
DeUgarte D et al	● (green)	● (green)	● (green)	● (green)	● (green)	● (green)	● (grey)	● (green)
Nemoto Y et al	● (green)	● (green)	● (green)	● (green)	● (green)	● (green)	● (grey)	● (green)
Ochi t et al	● (green)	● (green)	● (green)	● (green)	● (green)	● (green)	● (grey)	● (green)
Prasad A et al	● (green)	● (green)	● (green)	● (green)	● (green)	● (green)	● (grey)	● (green)
Hanke R et al.	● (green)	● (green)	● (green)	● (green)	● (green)	● (green)	● (grey)	● (green)
Fukushima et al	● (green)	● (green)	● (green)	● (green)	● (green)	● (green)	● (green)	● (green)
Kaneda S et al	● (green)	● (green)	● (green)	● (green)	● (green)	● (green)	● (grey)	● (green)
